# Glandular odontogenic cyst—Report of a rare case

**DOI:** 10.1002/ccr3.2638

**Published:** 2020-01-07

**Authors:** Pratibha Poudel, Ritesh Srii, Nitesh Chaurasia, Chandan Upadhyaya

**Affiliations:** ^1^ Department of Oral and Maxillofacial Pathology Kathmandu University School of Medical Sciences Dhulikhel Nepal; ^2^ Department of Oral and Maxillofacial Surgery Kathmandu University School of Medical Sciences Dhulikhel Nepal

**Keywords:** glandular, odontogenic cyst, sialo‐odontogenic cyst

## Abstract

Glandular odontogenic cyst is a rare developmental odontogenic cyst that bears similarity to several other odontogenic lesions. Till 2017, only 169 such cases have been reported in the literature. Herein, we describe one more case of it occurring in a 35‐year‐old female patient.

## Graphical abstract

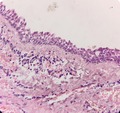

## 1 INTRODUCTION

Glandular odontogenic cyst (GOC), a rare entity among the developmental odontogenic cysts, was first recognized as sialo‐odontogenic cyst.^1^ In 1988, Gardner et al proposed the term “glandular odontogenic cyst” that was adopted by WHO in 1992.^2^ The odontogenic origin of this cyst has been confirmed by immunohistochemistry. It usually presents as an asymptomatic, slow‐growing mass in anterior segment of mandible and is seen mostly among the middle‐aged individuals with a slight increased predilection for males.^3^, ^4^

Radiologically, the lesion may appear unilocular or multilocular with defined borders which occasionally may show loss of cortical integrity and root resorption.^5^ Histologically, it is characterized by variable thickness of nonkeratinized epithelium consisting of superficial layer of cuboidal or columnar cells that are referred as hobnail cells, and occasionally, these cells may be ciliated.^6^ The epithelium might show papillary projections, nodular thickenings, microcystic areas, and mucous cells.^4^ It is imperative to make the correct diagnosis of this lesion as it is more aggressive, requires more radical surgery, and has higher chances of recurrence compared to other odontogenic cysts such as radicular cyst and dentigerous cyst. Herein, we report a case of GOC in a 35‐year‐old woman, which was radiologically mimicking the radicular cyst.

## 2 CASE REPORT

A 35‐year‐old woman presented to the Department of Oral and Maxillofacial Surgery, Dhulikhel Hospital, Dhulikhel, with the chief complain of asymptomatic swelling in the lower left back region of jaw since 2 months. On clinical examination, hard swelling was present on left body of mandible in relation to 36 and 37, obliterating the buccal vestibule and measuring about 1.5 cm in diameter. On palpation, the lesion was nontender, with no discharge, and the overlying mucosa was normal.

**Figure 1:**
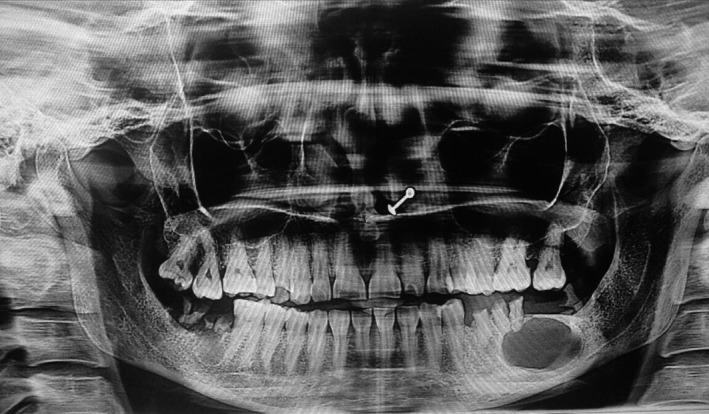
Orthopantomogram showing unilocular radiolucency involving root stumps of left mandibular first molar

**Figure 2:**
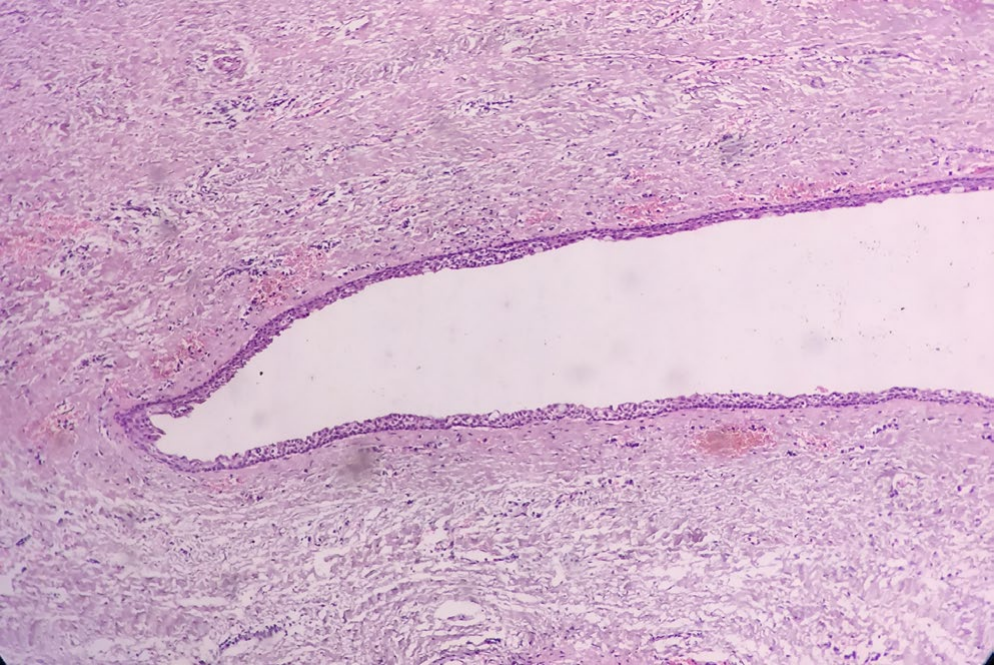
Cystic cavity lined by nonkeratinized stratified squamous epithelium that is focally ciliated. (H&E stain, ×100)

**Figure 3:**
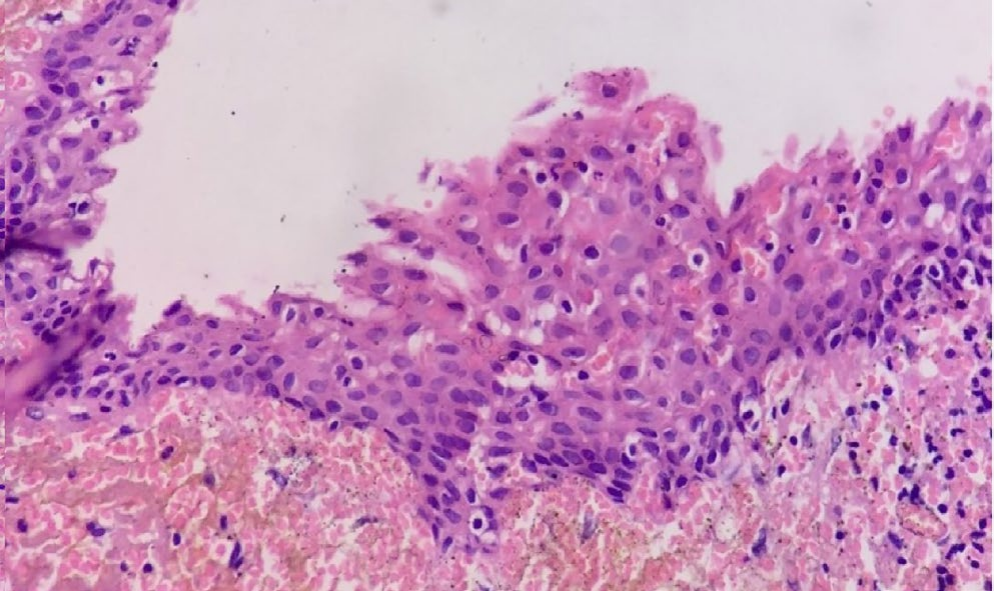
Lining epithelium showing plaque like thickening focally. (H&E stain, ×400)

**Figure 4:**
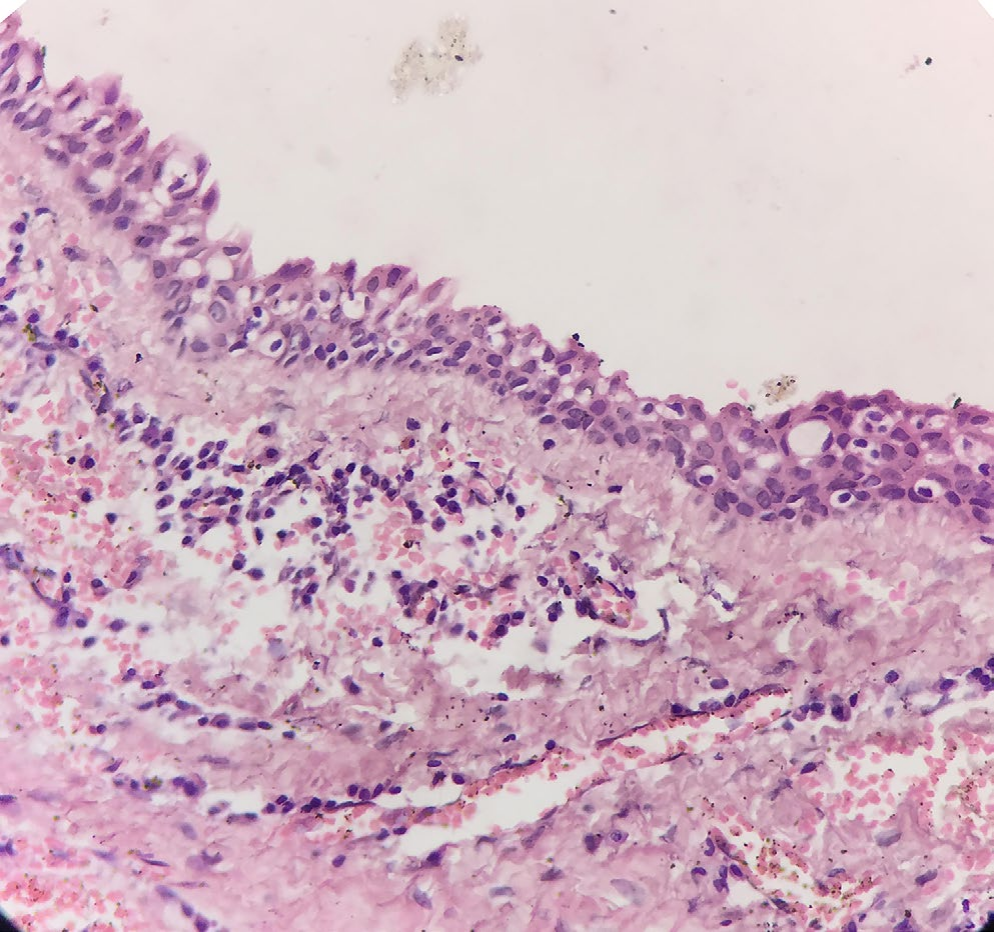
Lining epithelium showing hobnail cells, few clear cells, and microcystic space. (H&E stain, ×400)

**Figure 5:**
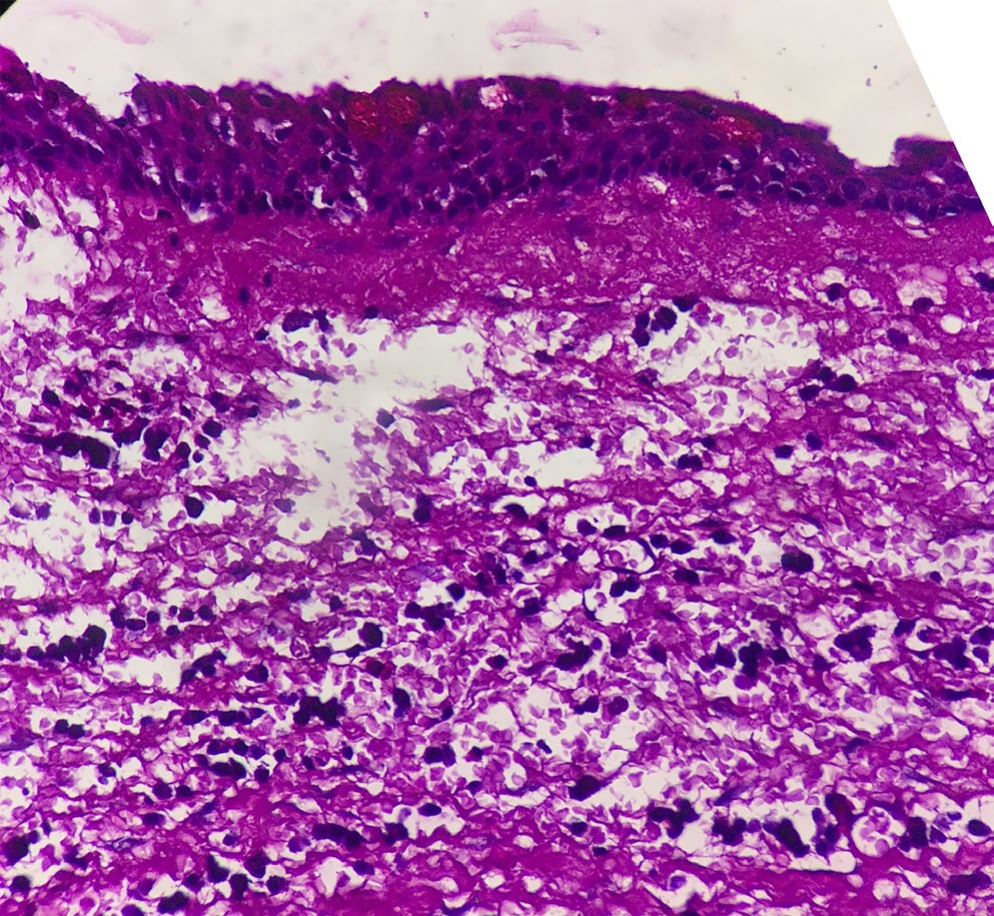
PAS stain showing mucous cells in the lining epithelium. (PAS stain, ×400)

Radiographic examination revealed a well‐defined unilocular radiolucency involving the body of mandible in relation to the apices of 36 with well‐defined corticated borders measuring 22.5 mm × 15.5 mm. Tooth numbers 35, 36, and 38 were nonvital, and 37 was missing (Figure 1). The root stumps of 35, 36, and 38 were extracted along with enucleation of cystic lining, and the specimen was sent for histopathological evaluation. Microscopic examination revealed cystic cavity lined by nonkeratinized stratified squamous epithelium, which was focally ciliated (Figure 2). The lining epithelium showed thickening/plaque formation at some places (Figure 3). The superficial layer of epithelium contained cuboidal to columnar eosinophilic cells resembling “hobnail” cells (Figure 4). PAS staining revealed numerous PAS‐positive mucous cells (Figure 5). Few duct‐like spaces were also noted in the epithelium. The connective tissue capsule showed dense collagen fibers, moderate chronic inflammatory cell infiltrate, and numerous extravasated RBC's. Based on these histopathological observations, the diagnosis of GOC was given.

## 3 DISCUSSION

Glandular odontogenic cyst is a rare developmental odontogenic cyst accounting for about 0.012% to 1.3% of all jaw cysts.^4^ Till 2017, only 169 cases of GOCs have been reported. Chrcanovic et al did an extensive review of these 169 cases and found that these lesions were most common in males compared to females with predilection for anterior mandible.^7^ In contrast to this study, our case favored female predilection and the lesion was seen in posterior mandible rather than the anterior region. Only two cases of GOCs have been previously reported from Nepal, and both cases were seen in maxilla.^8^

The lesion is usually asymptomatic,^9^ and this was true in our case as well. Radiographically, the features of GOC are variable and are not pathognomonic.^10^ It may present as unilocular or multilocular radiolucency with well‐defined borders.^11^ Our case exhibited unilocular radiolucency with well‐defined borders involving the roots of 36, thus mimicking the radicular cyst. Studies have shown that radiographically, GOC mimics several other lesions such as radicular cyst, central giant cell granuloma, ameloblastoma, and keratocyst.^10^, ^12^

As both clinical and radiological features of GOC are deceptive, histopathological examination of the lesion becomes crucial to arrive at the final diagnosis. However, this also poses a diagnostic challenge, as its histopathological features resemble other lesions such as lateral periodontal cyst (LPC), botryoid odontogenic cyst (BOC), and central mucoepidermoid carcinoma (CMEC). Several authors have put forward the microscopic criteria's for diagnosing of GOC. Kaplan et al established major and minor criteria for diagnosis of GOC, whereas Fowler et al proposed ten specific microscopic features and suggested that the presence of seven or more of these features are highly predictive of GOC.^1^ The present case shows most of these characteristic features such as nonkeratinized stratified squamous epithelium, epithelial whorls or focal luminal proliferation, superficial cuboidal or eosinophilic cells referred as “hobnail cells” which are occasionally ciliated, clear or vacuolated cells in basal or spinous layer, and presence of mucous cells with microcystic areas. The presence of ciliated cells, duct‐like spaces, and mucous cells differentiates GOC from LPC and BOC.^13^ Nonetheless, differentiating GOC from low‐grade CMEC is more difficult; the latter lacks hobnail cells, epithelial whorls, ciliated cells, and intraepithelial microcyst formation. Adjunctive tools such as cytokeratin (CK) profiles can also be helpful in difficult situation. CMEC expresses CK 18 where as GOC expresses CK 19.^13^, ^14^

The treatment modalities of GOC include enucleation with or without curettage, marsupialization, peripheral ostectomy, chemical cauterization with carnoy's solution, and marginal or segmental resection.^9^ Nevertheless, conservative surgical approach such as enucleation with or without curettage and peripheral ostectomy are the commonest treatment modalities reported in the literature.^15^, ^16^ Due to its tendency to recur after conservative surgical approach, some authors prefer marginal resection and segmental resection especially in larger lesions. The high rate of recurrence can be attributed to thin lining, multilocularity of cyst, presence of microcysts, and high mitotic capacity of cells similar to odontogenic keratocyst.^17^ Some authors have also reported that larger lesions that have perforated the cortical bone and are radiographically multilocular have more chances to recur. These lesions would be amenable to radical approach such as marginal or segmental resection with or without resection of overlying mucosa.^18^ According to Kaplan et al if the cyst is small and completely enucleated, further surgery is not indicated, as recurrence is unlikely in these cases. Nevertheless, patient should be kept under follow‐up for a minimum of 3 years.^19^ In the present case, as the lesion was small, the cyst was completely enucleated and the final diagnosis was evident only after the histopathological examination. The patient has been kept under regular follow‐up, and in case of any clinical or radiographic signs of recurrence, radical approach such as marginal or segmental resection with or without resection of overlying mucosa will be performed.

## 4 CONCLUSION

Glandular odontogenic cyst is a rare odontogenic cyst that has aggressive clinical behavior and high rate of recurrence. Due to its nonspecific clinico‐radiographic features, it is often misdiagnosed. Hence, careful histopathological evaluation is necessary for final diagnosis along with the long‐term follow‐up to rule out the recurrences.

## CONFLICT OF INTEREST

None declared.

## INFORMED CONSENT

Informed consent was obtained from the patient for publication of the case.

## AUTHOR CONTRIBUTIONS

PP: is a primary author, prepared, structured, and formatted case report. RS, NC, and CU: critically reviewed the manuscript.
